# Coinfection of intestinal schistosomiasis and malaria and association with haemoglobin levels and nutritional status in school children in Mara region, Northwestern Tanzania: a cross-sectional exploratory study

**DOI:** 10.1186/s13104-017-2904-2

**Published:** 2017-11-09

**Authors:** Safari M. Kinung’hi, Humphrey D. Mazigo, David W. Dunne, Stella Kepha, Godfrey Kaatano, Coleman Kishamawe, Samuel Ndokeji, Teckla Angelo, Fred Nuwaha

**Affiliations:** 1 National Institute for Medical Research, Mwanza Centre, P. O. Box 1462, Mwanza, Tanzania; 2 Department of Medical Parasitology and Entomology, School of Medicine, Catholic University of Health and Allied Sciences, P. O. Box 1464, Mwanza, Tanzania; 3 Department of Pathology, Cambridge University, Tennis Court Road, Cambridge, UK; 4 Kenya Medical Research Institute (KEMRI), Nairobi, Kenya; 5 Department of Disease Control and Environmental Health, School of Public Health, Makerere University, P. O. Box 7072, Kampala, Uganda

**Keywords:** Coinfection, Schistosomiasis, Malaria, Mara region, Tanzania

## Abstract

**Background:**

Schistosomiasis represents a major public health problem in Tanzania despite ongoing national control efforts. This study examined whether intestinal schistosomiasis is associated with malaria and assessed the contribution of intestinal schistosomiasis and malaria on anaemia and undernutrition in school children in Mara region, North-western Tanzania.

**Methods:**

Stool samples were collected from each of 928 school children randomly selected from 5 schools and examined for intestinal schistosomiasis using the Kato Katz method. Finger prick blood samples were collected and examined for malaria parasites and haemoglobin concentrations using the Giemsa stain and Haemocue methods, respectively. Nutritional status was assessed by taking anthropometric measurements.

**Results:**

The overall prevalence and infection intensity of *S. mansoni* was 85.6% (794/928) and 192 (100–278), respectively. The prevalence of malaria was 27.4% (254/928) with significant differences among villages (χ^*2*^ = 96.11, p < 0.001). The prevalence of anaemia was 42.3% (392/928) with significant differences among villages (χ^*2*^ = 39.61, p < 0.001). The prevalence of stunting, thinness and underweight was 21, 6.8 and 1.3%, respectively. Stunting varied significantly by sex (χ^*2*^ = 267.8, p < 0.001), age group (χ^*2*^ = 96.4, p < 0.001) and by village (χ^*2*^ = 20.5, p < 0.001). Out of the 825 infected children, 217 (26.4%) had multiple parasite infections (two to three parasites). The prevalence of co-infections occurred more frequently in boys than in girls (*χ* ^*2*^ = 21.65, p = 0.010). Mean haemoglobin concentrations for co-infected children was significantly lower than that of children not co-infected (115.2 vs 119.6; t = 0.01, p = 0.002). Co-infected children were more likely to be stunted than children who were not co-infected (χ^*2*^ = 11.6, p = 0.003). On multivariate analysis, age group, village of residence and severe anaemia were significant predictors of stunting after adjusting for sex and infection status.

**Conclusions:**

Intestinal schistosomiasis and malaria are prevalent in Mara region. Coinfections of these parasites as well as chronic undernutrition were also common. We recommend Mara region to be included in national schistosomiasis control programmes.

## Background

Schistosomiasis represents a major public health problem globally [1–4]. Of the world’s 207 million estimated cases of schistosomiasis, more than 90% occur in sub-Saharan Africa (SSA) [3]. In Tanzania both intestinal schistosomiasis (caused by *Schistosoma mansoni*), and urogenital schistosomiasis (caused by *Schistosoma haematobium*) are prevalent particularly in school age children, adolescents and in fishing communities living along the shores of Lake Victoria where prevalence of infection higher than 90% are common [5, 6]. The country is ranked second in terms of burden of schistosomiasis in sub-Saharan Africa, the first being Nigeria [3]. Malaria caused by *P. falciparum* also occurs throughout the country [7, 8]. Mara region, on the Northwestern part of Tanzania has long been considered a high transmission area for both schistosomiasis and malaria due to its geographical location in the Lake Victoria basin which is highly endemic for both diseases [6, 9, 10]. As a result of geographical overlap between schistosomiasis and malaria, coinfections of these parasites are common which results into various forms of associations, exacerbated health consequences and co-morbidities [11–15]. Evidence from epidemiological studies have indicated that individuals co-infected with more than one parasite species have increased susceptibility to other infections [16–19] and at a risk of developing frequent and more severe disease due to interactions among the infecting parasite species [11, 16, 17, 20–23]. Anaemia and undernutrition are among the most common conditions observed in many field studies in sub-Saharan Africa, Tanzania inclusive, and parasitic infections are among major causes [10, 24–31]. Mechanisms through which parasitic infections cause anaemia and undernutrition include damage of intestinal mucosa which in turn result into impaired digestion and absorption of nutrients [30–32], direct destruction and/or intestinal blood loss [33–36], interference with processes leading to production of blood cells in the bone marrow [30] and impaired immune development [37–39]. In school aged children, health consequences associated with parasitic infections include suboptimal physical development and reduced learning and school achievements [11, 30, 31]. Despite the recognition of parasitic infections as important causes of anaemia and undernutrition in human populations in endemic countries, their combined effect on human health has not being thoroughly investigated [26, 40, 41]. More research is therefore needed to gain a better understanding of the burden of co-infections, describe their combined health consequences on affected populations and define their implication on disease control strategies [42, 43]. The current study examined whether infection with intestinal schistosomiasis is associated with malaria infection and assessed the contribution of intestinal schistosomiasis and malaria on anaemia and undernutrition in school children in Mara region, North-western Tanzania.

## Methods

### Study area and population

**Fig. 1:**
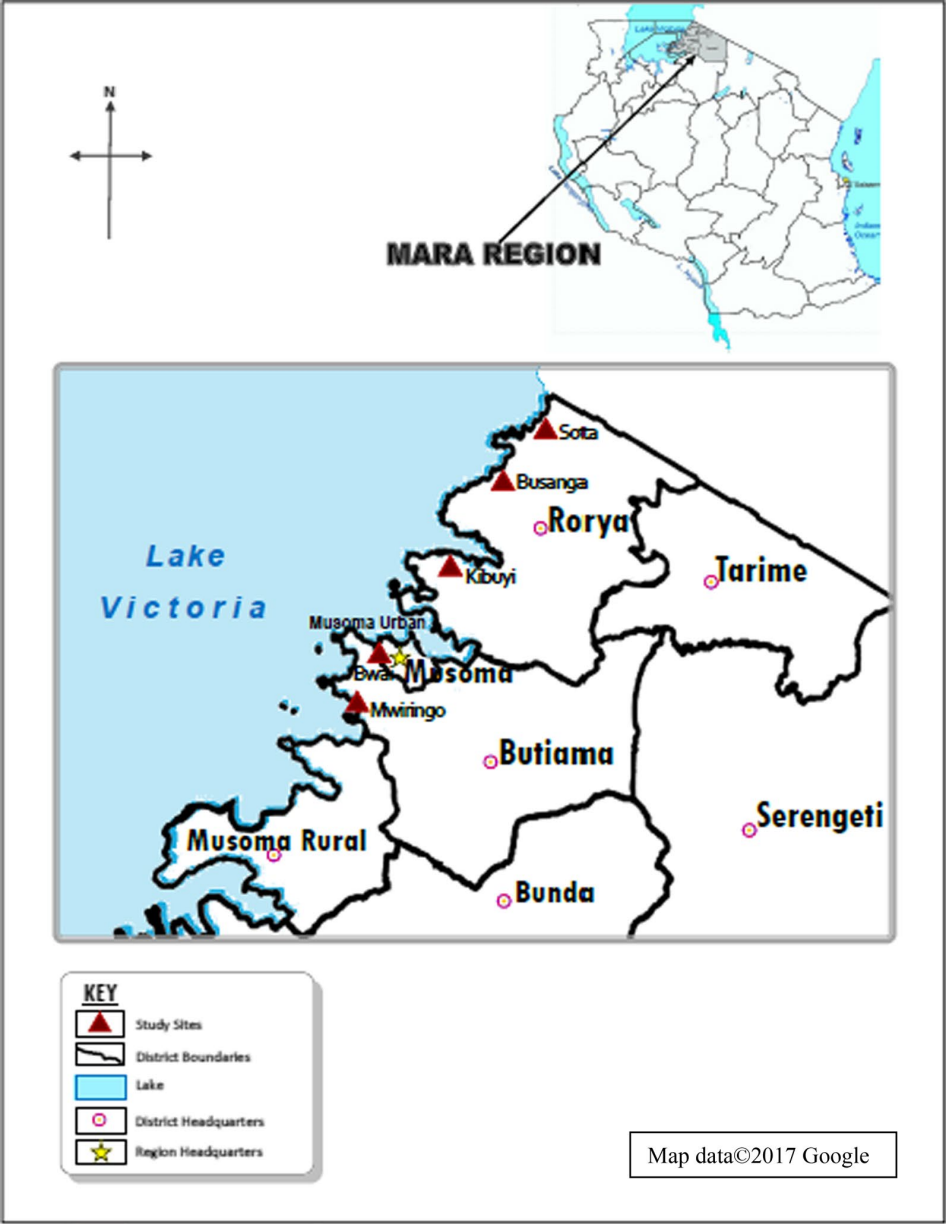
Map of Tanzania showing Rorya and Butiama districts and the study villages in each district (Adapted from google: https://www.google.com/search?q=tanzania+maps)

The study was conducted during the dry season (September) of the year 2013, in Mara region, Northwestern Tanzania. Mara region lies between latitudes 1°0′ and 2°31′ South of the Equator and between longitudes 33°1′ and 35°15′ east of Greenwich meridian. The region has six districts namely Serengeti, Tarime, Bunda, Musoma, Butiama and Rorya. The region lies within the Lake Victoria basin and has a maximum temperature of 29.3 °C and a minimum temperature of 27.7 °C while the average temperature is 28.5 °C. The region has bimodal rainfall pattern with short rainfall period between September and January and long rainfall period between February to June each year. Average annual rainfall varies from 1250 to 2000 mm. The predominant ethnic groups in the region include the Kurya, Simbiti, Luo and Jita among others who practise subsistence farming (animal husbandry and crops) and fishing in Lake Victoria [44]. Schistosomiasis and soil-transmitted helminth infections rank number five as the most common causes of morbidity in the region after malaria, upper respiratory tract infections (URTIs), diarrhoeas and pneumonias in that order [45]. Two districts of Rorya and Butiama both lying along the Eastern shores of the Lake Victoria were purposively selected for this study because their land mass borders the eastern shores of Lake Victoria and haven’t been involved in larger scale public health interventions targeting schistosomiasis and soil-transmitted helminth infections. The population of Rorya and Butiama districts is 265,241 and 241,732, respectively [45]. From the two districts, a total of five villages were conveniently selected from among villages closely located along the lake shore. Selection was based on easy of accessibility and support from teachers, village leaders and parents. For Rorya district three villages were selected namely Sota, Busanga, and Kibuyi. For Butiama district two villages were selected namely Mwiringo and Bwai (Fig. 1).

Each of the selected villages had at least one primary school. The mean school enrolment rate was 840 (range 590–1289). From each selected village, school children were selected and included in the study. Based on World Health Organization (WHO) recommendations [32], in order to evaluate for prevalence and intensity of helminth infections, a sample size of about 200 school children was required for each participating village.

### Study design, sampling procedures, inclusion and exclusion criteria

The study was a cross-sectional exploratory parasitological survey utilizing quantitative data collection methods. School children were selected from grades 1–6 whereby about 30–35 children were selected from each grade using systematic random sampling procedure while balancing for age and sex. Briefly, children in each grade were requested to stand in two lines, one for boys and one for girls and they were counted. For each sex, the sampling interval was calculated by dividing the total number of children in the line with the number of children to be examined. After obtaining a starting point, children were sampled according to the sampling interval until the required number of children for each sex in each grade was obtained. Children in grade seven were not included because they were about to do their final national examinations and hence it was decided not to disturb them. The inclusion criteria were: Being school children (age 6–15 years) living in the selected villages for at least 1 year. In addition, selected children were those who provided informed accent and informed consent from their parents or guardians. Exclusion criteria were: Children who had stayed in the village for less than 1 year, children who did not provide informed accent and informed parental/guardian consent.

### Data collection methods

#### Parasitological examination for schistosomiasis, soil-transmitted helminth infections and malaria

From all selected school children, two stool samples were collected on two consecutive days from which four Kato Katz thick smears were prepared and examined for intestinal schistosomiasis and other helminth infections. Microscopic examination was performed by well trained and experienced laboratory technicians. Examination for hookworm infections was performed within 1 h of smear preparation while examination for *S. mansoni*, *Ascaris lumbricoides* and *trichuris trichiura* was performed at least 24 h after smear preparation. *S. mansoni* infections was classified as light (epg < 100), moderate (epg 100–399) and heavy (epg ≥ 400). About 10 mL of urine samples were also collected from the same participants and examined for urinary schistosomiasis using the urine filtration method. In addition, fingerpick blood was collected and examined for malaria parasitaemia and haemoglobin concentrations using the Giemsa stain and HemoCue methods, respectively. The number of malaria parasites per µL of blood was calculated assuming 8000 white blood cells/µL of blood. The number of malaria parasites was counted per 200 WBC and transformed into malaria parasite density by multiplying by a factor of 40. Malaria parasite density was classified as low (< 5000 parasites/µL of blood) and high (≥ 5000 parasites/µL of blood). A blood slide was considered negative if no parasites were observed after counting 100 fields. Anaemia was defined according to WHO guidelines and adjusted for age and sex [46]. Nutritional status was determined by taking anthropometric measurements i.e. weight (measured using a digital weight scale to the nearest 0.1 kg) and height (measured using a portable height pole to the nearest 0.1 cm). Age was obtained from school registers and recorded in years.

#### Data management and analysis

All data collected was managed by the data management unit of the National Institute for Medical Research (NIMR), Mwanza centre. Data were double entered into the Census and Survey Processing System (CSPro) software (US Census Bureau, USA) and analyzed using STATA Version 12 (STATA Corp., Texas, USA). Infection intensities (of positive samples only) were calculated as geometric mean of parasites per microlitre of blood for *P. falciparum* and geometric mean of eggs per gram of faeces for *S. mansoni,* hookworms, *Ascaris lumbricoides* and *trichuris trichiura*. For *S. haematobium* infection, infection intensity was calculated as geometric mean eggs/10 mL of urine. Nutritional status was expressed as stunting (height-for-age) and wasting [body mass index (BMI)-for-age] and underweight (weight for age). Data on nutritional status was analysed by converting anthropometric measurements into height-for-age Z scores (HAZ) and body mass index (BMI) Z scores (BAZ) and weight for age Z scores (WAZ) using WHO guidelines (Anthro Plus version 1.0.4). Children whose HAZ, BAZ and WAZ fell below 2 standard deviations were considered thin, stunted or wasted, respectively. The student’s *t* test and one way analysis of variance (ANOVA) were used to compare geometric mean parasite counts and mean haemoglobin concentrations between groups where two or more than two groups were compared, respectively. The Chi square test was used to compare proportions (prevalence) of *S. mansoni* infection, malaria infection and prevalence of anaemia between villages and other socio-demographic characteristics. Multivariate logistic regression analysis was used to assess for predictors of anaemia and undernutrition (stunting). The model for anaemia was adjusted for age, sex, village of residence and co-infection status while the model for stunting was adjusted for sex and co-infection status. The Hosmer–Lemeshow (HL) test for goodness of fit (GOF) was used to test for goodness of fit for the multivariate logistic regression models for anaemia and stunting. All graphs were drawn using MS excel. Tests were considered statistically significant at p < 0.05.

## Results

A total of 928 (93.3%) children had complete parasitological and anthropometric data and were included in this analysis. The distribution of the 928 children with respect to the five villages was: Sota (n = 179), Busanga (n = 183), Kibuyi (n = 171), Bwai (n = 196) and Mwiringo (n = 199). Boys were 445 (47.9%). Median age was 11 years (range 6–15 years). The overall baseline and demographic characteristics of the studied population are shown in Table 1.

**Table 1:** Description of the baseline and demographic characteristics of the studied population by village (n = 928)

Characteristic	Village					p value
	Sota	Busanga	Kibuyi	Mwiringo	Bwai	
Sex						
Boys (n, %)	76 (17.1)	93 (20.9)	100 (22.4)	103 (23.2)	73 (16.4)	
Girls (n, %)	103 (21.3)	90 (18.6)	71 (14.7)	96 (19.8)	123 (25.5)	< 0.001
Age group (years)						
6–8 (n, %)	36 (20.1)	53 (29.0)	46 (26.9)	63 (31.7	70 (35.7)	
9–12 (n, %)	96 (53.6)	91 (49.7)	82 (48.0)	80 (40.2	99 (50.5)	
13–15 (n, %)	47 (26.3)	39 (21.3)	43 (25.2)	56 (28.1	27 (13.8)	0.003
*S. mansoni* prevalence (%)	164 (91.6)	154 (84.2)	160 (93.6)	126 (63.3)	190 (96.9)	< 0.001
*S. mansoni* intensity (epg)	118.1 (94.3–147.8)	204.5 (164.5–254.2)	187.3 (150.4–233.3)	72.2 (51.3–94.2)	349.3 (295.2–413.3)	< 0.001
Hookworm prevalence (%)	6 (3.3)	17 (9.3)	4 (23.0)	13 (6.5)	0	< 0.001
Hookworm intensity (epg)	147.8 (13.4–1623.8)	96.0 (45.3–203.7)	51.6 (19.6–135.6)	161.7 (105.4–258.1)	–	0.210
Malaria prevalence (%)	50 (27.9)	90 (49.2)	47 (27.5)	58 (29.2)	9 (4.6)	< 0.001
Malaria parasite density (mps/µL)	1097.9 (712.8–1691.0)	591.3 (448.1–780.3)	1608.2 (1020.6–2534.2)	2136.5 (1472.6–3099.7)	609.6 (160.1–2321.2)	< 0.001
Prevalence of co-infection (%)	47 (26.3)	73 (39.9)	51 (29.8)	37 (18.6)	9 (4.6)	< 0.001
Anaemia prevalence (%)	96 (53.6)	82 (44.8)	71 (41.5)	59 (29.7)	84 (42.8)	< 0.001
Mean Hb (g/dL)	111.2 (107.8–114.6)	115.5 (112.7–118.4)	119.5 (116.9–121.9)	125.2 (123.5–126.4)	120.6 (118.1–123.0)	< 0.001
Prevalence of stunting (%)	29 (16.2)	47 (25.6)	44 (25.7)	52 (26.1)	23 (11.7)	< 0.001
Prevalence of thinness (%)	15 (8.4)	8 (4.4)	18 (10.5)	18 (9.1)	4 (2.0)	0.005
Prevalence of underweight (%)	2 (1.1)	3 (1.6)	2 (1.2)	2 (1.0)	3 (1.5)	0.998

### Prevalence and infection intensity of intestinal schistosomiasis and other helminth infections

Out of the 928 children with complete information 825 (88.9%) were infected by at least one of the parasites *S. mansoni*, *S. haematobium*, hookworm, *Trichuris trichiura, Ascaris lumbricoides* and malaria. The prevalence of each parasite species was as follows: *S. mansoni* (85.6%, range 63.3%–96.9%), *S. haematobium* (0.8%, range 0%–2.3%), hookworm (4.3%, range 0%–9.3%), *T. trichiura* (0.2%, range 0%–0.5%) and *A. lumbricoides* (0.6%, range 0%–3.4). The infection intensities (with 95% confidence intervals) of each parasite species was as follows: *S. mansoni* (192, 100–278), *S. haematobium* (11, 4–42), hookworm (105, 57–248), *T. trichiura* (24, 6–48) and *A. lumbricoides* (498, 87–2856). Out of the 928 school children with complete parasitological data for *S. mansoni* infection, 251 (27%) had light infections, 296 (31.9%) had moderate infections and 247 (26.6%) had heavy infections. Intestinal schistosomiasis was more prevalent in all villages while infections with other helminth species occurred at very low levels.

The prevalence and infection intensity of *S. mansoni* in school children varied significantly from village to village and among age groups (p < 0.001) (Table 1). However, there was no significant variation in prevalence and infection intensity between sexes (p > 0.05).

### Prevalence of malaria and anaemia and associations with *S. mansoni* infection

The prevalence of malaria in school children was 27.4% (254/928) (range 4.6-49.2%) out of whom 6.1% had heavy infections (≥ 5000/µL of blood). The prevalence of malaria varied significantly among villages (χ^*2*^ = 96.11, p < 0.001) and between sexes (χ^*2*^ = 11.28, p < 0.01). There was no significant differences in malaria prevalence between age groups (χ^*2*^ = 0.2690, p = 0.874). Mean malaria parasite density was 1086.7 (899.6–1312.5) with significant differences among villages. The prevalence of anaemia was 42.3% (392/928) (range 29.7–53.6%) with significant differences among villages (Table 1). The prevalence of severe anaemia was 3.1% (29/928). The prevalence of anaemia did not differ significantly between sexes or between age groups (p > 0.05). Mean haemoglobin concentration was 118.8 (117.6–120.0) with significant differences among villages (F = 17.2, p < 0.001). The prevalence of anaemia was higher among children with malaria infection (χ^*2*^ = 8.77, p = 0.012) and those with *S. mansoni* infection (χ^*2*^ = 13.0, p < 0.01) than in children without these infections.

**Fig. 2:**
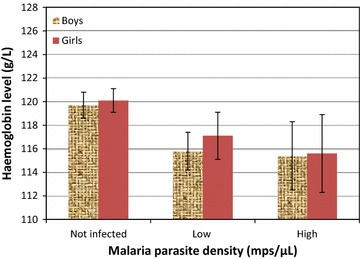
Association between haemoglobin levels and malaria parasite density in school children (n = 928)

Children with malaria infection had lower mean haemoglobin levels compared to un-infected children. Haemoglobin concentrations decreased significantly with increasing malaria parasite density (F = 4.98, p < 0.01) (Fig. 2).

**Fig. 3:**
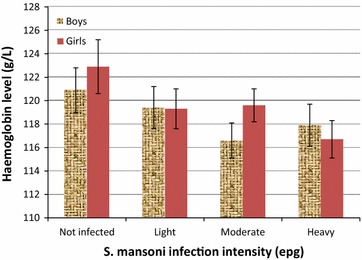
Association between haemoglobin levels and *S. mansoni* infection intensity in school children (n = 928)

Children with *S. mansoni* infection had lower mean haemoglobin levels compared to un-infected children (Fig. 3). Haemoglobin concentrations decreased with increasing *S. mansoni* infection intensity. However, this effect was not statistically significant (F = 2.14, p = 0.093) (Fig. 3). On multivariate logistic regression analysis and adjusting for possible confounders, malaria parasite density and *S. mansoni* infection intensity remained to be significant predictors of anaemia. For malaria parasite density, this effect was more marked for children with low malaria parasite density who were 1.6 times more likely to be anaemic compared to un-infected children and this effect was statistically significant (p = 0.003) (Table 2). For *S. mansoni* infection intensity, this effect increased with increasing infection intensity whereby children with moderate to heavy infection intensity were about 2.0 times more likely to be anaemic compared to un-infected children and this effect was statistically significant (p < 0.01) (Table 2).

**Table 2:** Multivariate logistic regression analysis of predictors of anaemia in school children in the studied population (n = 928)

Independent variable	Categories	Adjusted OR (CI)	p value
Sex	Female	1	
	Male	1.103 (0.844–1.442)	0.471
Age group (years)	6–8	1	
	9–12	1.067 (0.781–1.442)	0.683
	13–15	0.777 (0.533–1.133)	0.190
Malaria infection	Not infected	1	
	Low infection	1.632 (1.182–2.255)	0.003
	High infection	1.307 (0.752–2.271)	0.342
*S. mansoni* infection	Not infected	1	
	Light infection	1.337 (0.851–2.100)	0.207
	Moderate infection	1.870 (1.207–2.898)	0.005
	Heavy infection	2.075 (1.322–3.257)	0.002
Stunting	Normal	1	
	Stunted	1.232 (0.813–1.867)	0.323
Thinness	Normal	1	
	Thin	1.052 (0.602–1.837)	0.859
Underweight	Normal	1	
	Underweight	1.596 (0.484–5.262)	0.442

### Prevalence of co-infections and association with anaemia and undernutrition

Out of the 825 infected children, 608 (73.6%) had single parasite infections while the rest 217 (26.4%) had multiple parasite infections (two to three parasites). The most common single parasite infection was *S. mansoni* (67.7%) followed by *P. falciparum* malaria (5.2%) and hookworm (0.6%). The most common multiple parasite infection was that of *P. falciparum* and *S. mansoni* which accounted to 88.4% of all co-infections. Out of the 254 children who were infected with *P. falciparum*, 208 (81.9%) were concurrently infected with *S. mansoni*. However, there was no significant association between *S. mansoni* and *P. falciparum* infections (*χ* ^2^ = 3.81, p = 0.051). Other parasite pairs occurred less frequently and included *S. mansoni* and hookworm (1.9%), *S. mansoni* and *S. haematobium* (0.6%) and hookworm and malaria (0.4%). The prevalence of co-infections occurred more frequently in boys than in girls (*χ* ^*2*^ = 21.65, p = 0.010). Mean haemoglobin concentrations for children who were co-infected with more than one parasite was 115.2 (112.7–117.5) and was significantly lower than mean haemoglobin concentrations for children who were not co-infected (119.6, 95% CI 118.1–121.0) (t = 0.01, p = 0.002). Surprisingly, the prevalence of severe anaemia was significantly higher in children with single parasite infection than in children with more than one parasite infection (*χ* ^*2*^ = 18.36, p < 0.01). The prevalence of stunting, thinness and underweight was 21, 6.8 and 1.3%, respectively. On bivariate analysis, stunting varied significantly by sex (χ^*2*^ = 267.8, p < 0.001), age group (χ^*2*^ = 96.4, p < 0.001) and by village (χ^*2*^ = 20.5, p < 0.001) whereby boys were more likely to be stunted than girls and older children (13–16 years) were more likely to be stunted than young children. Further, co-infected children were more likely to be stunted, than children who were not coinfected (χ^*2*^ = 11.6, p = 0.003). Anaemic children tended to be stunted but this relationship did not reach statistical significance (χ^*2*^ = 2.48, 0.290). On multivariate logistic regression analysis, only age group, village of residence and severe anaemia remained to be significant predictors of stunting after adjusting for sex and co-infection status. Compared to children in the age group of 6–8 years, children in the age group of 9–12 years were 12.3 times more likely to be stunted while children in the age group of 13–15 years were 23.7 times more likely to be stunted (Table 3). Children with severe anaemia were about 17.2 times more likely to be stunted compared to normal (non-anaemic) children (Table 3).

**Table 3:** Multivariate logistic regression analysis of predictors of stunting, in school children in the studied population (n = 928)

Independent variable	Categories	Adjusted OR (CI)	p value
Age group (years)	6–8	1	
	9–12	12.30 (5.59–26.9)	< 0.001
	13–15	23.70 (10.6–53.3)	< 0.001
Village	Busanga	1	
	Bwai	0.397 (0.190–0.827)	0.014
	Kibuyi	0.712 (0.362–1.399)	0.325
	Mwiringo	1.130 (0.562–2.257)	0.729
	Sota	0.346 (0.169–0.704)	0.003
Malaria infection	Not infected	1	
	Low infection	0.719 (0.409–1.266)	0.254
	High infection	0.632 (0.264–1.515)	0.304
*S. mansoni* infection	Not infected	1	
	Light infection	0.550 (0.242–1.253)	0.156
	Moderate infection	0.838 (0.365–1.921)	0.677
	Heavy infection	1.036 (0.435–2.464)	0.936
Anaemia	Normal	1	
	Anaemic	0.946 (0.598–1.494)	0.811
	Severely anaemic	17.160 (2.632–34.688)	0.003
Coinfection	Not infected	1	
	Single infection	0.961 (0.388–2.411)	0.934
Coinfection	Double/triple infection	2.077 (0.623–6.921)	0.234

The Hosmer–Lemeshow (HL) test for goodness of fit (GOF) showed that the multivariate logistic regression models for anaemia (Table 2) and stunting (Table 3) were a good fit (HL χ^*2*^ = 11.09, df = 8, p = 0.196) and (HL χ^*2*^ = 2.54, df = 8, p = 0.959), respectively. On the other hand, thinness was significantly associated with sex, age group and malaria infection. Boys were more likely to be thin than girls (χ^*2*^ = 73.36, p < 0.001) whereas older children were more likely to be thin than young children (χ^*2*^ = 24.10, p < 0.001) and malaria infected children were more likely to be thin than those not infected (χ^*2*^ = 3.91, p = 0.048). However, on multivariate analysis, only age group remained to be a significant predictor of thinness after adjusting for sex, village of residence and infection status (OR = 6.98, p < 0.001).

## Discussion

This research article reports findings of a cross-sectional exploratory study conducted in school children in five villages of Mara region, North-western Tanzania, an under researched area for schistosomiasis and other parasitic infections. The findings show that intestinal schistosomiasis is highly prevalent in school children in the study villages. All villages surveyed had a prevalence of infection above the WHO threshold of 50% for high transmission [47] indicating that Mara region is a high transmission area for intestinal schistosomiasis. As expected, both the prevalence and infection intensity was high in school children indicating that the risk of infection was also high for the rest of the population. These observations are consistent with previous studies conducted in other areas of the lake Victoria basin where high prevalence of *S. mansoni* was reported [10, 15, 48, 49] and highlights the importance of directing control efforts to the entire community. The prevalence of hookworm and other helminth infections *Ascaris lumbricoides* and *Trichuris trichiura* was very low also in line with previous studies [10, 15, 50]. Reasons for this are unknown but previous studies have suggested environmental factors such as soil types, temperature and humidity which influence survival of infective larvae [6, 9]. The prevalence of *S. haematobium* was very low probably due to the fact that the study was conducted in villages along the shore line where *S. haematobium* transmission rarely occurs [10, 15, 51]. The prevalence and infection intensity of intestinal schistosomiasis varied by age, being more prevalent in older school children than young children which is a normal pattern reflecting water contact behaviour and susceptibility to infection in relation to age and acquisition of natural immunity [52–54]. Further, the prevalence and infection intensity varied from village to village probably due to the focal nature of the disease depending on availability of infected snail intermediate hosts and hence varying patterns of exposure to water contaminated by schistosome cercariae. Contrary to what is already established [10, 55–59], for all age groups, sex of participants was not found to be associated with the risk of schistosomiasis by this study implying that the risk of infection in the studied population did not differ between boys and girls. The reason for this observation was not clear but it could be due to the fact that water contact patterns and hence the risk of infection did not differ significantly by sex. Malaria was also prevalent in all study villages and substantial level of co-infections existed between intestinal schistosomiasis and malaria. It is not surprising that high prevalence of anaemia was observed in the study villages and was significantly associated with high infection levels of the two parasites *P. falciparum* and *S. mansoni* as previously reported by other studies [10, 15, 48]. Although the etiology of anaemia is always multifactorial, this study has provided further evidence that parasitic infections are frequently associated with anaemia as it has been observed by previous studies [10, 15, 48, 55, 60]. The observed variations in the prevalence of anaemia between the current study and previous studies could be explained by changing pattern of both levels of anaemia and of helminth infections. Another possible explanation could be due to differences in age distribution of children who participated in the different studies. Contrary to intestinal schistosomiasis where anaemia was associated with heavy parasite loads, participants with low level of malaria parasitaemia were more likely to be anaemic compared to those with high level of malaria parasitaemia showing the importance of low level parasitaemia as a predictor of anaemia. Ezeamama et al. made a similar observation whereby children with low levels of parasite infections were more likely to be severely anaemic compared to uninfected children. Low levels of parasite loads represent chronic parasite infections which may play a major role in clinical morbidity [40]. Apart from acting concurrently as the major predictors of anaemia, *S. mansoni* and *P. falciparum* infections were found to be not associated with each other in this study, contrary to findings of other studies [17, 60, 61] which observed positive associations between the two parasites and attributed the observed associations to immunological interactions. Stunting was the most prevalent form of undernutrition reflecting the magnitude of chronic undernutrition and impaired childhood development in the studied population. Previous similar studies in East Africa [10, 14, 36] also observed high levels of undernutrition and attributed these to chronic parasitic infections and anaemia. The other two forms of undernutrition thinness and underweight occurred less frequently also in line with previous studies [26], indicating that they are of no public health significance in the studied population. Stunting was significantly associated with age and severe anaemia probably due to the fact that older children have been exposed to chronic undernutrition for a longer time period compared to younger children. The same could be the case for anaemia whereby anaemic children could be more exposed to chronic undernutrition and infections compared to non-anaemic children. This observation is in line with findings of other studies [26, 38, 62, 63], but needs further investigation. On the other hand, stunting varied significantly from village to village indicating the importance of micro-geographical variations from one village to the next. This study did not observe a significant association of single or multiple parasitic infections and undernutrition. Although an association between single and multiple parasite infections and undernutrition have being reported by other studies [14, 63], this association remains unclear as other studies report no association [26, 27, 64]. The lack of association between single and multiple parasitic infections and undernutrition in this study could probably be due to presence of other factors such as socio-economic status and other infections which might be associated with chronic undernutrition and hence they acted as confounders. The lack of information on these factors was a limitation of the current study which makes it difficult to draw firm conclusions from observed findings. The cross-sectional design of the study was another limitation as this also does not allow firm conclusions on causation to be made.

## Conclusions

From these findings, it could be concluded that high transmission of intestinal schistosomiasis and malaria occurs in Mara region like other regions in the Lake Victoria basin. Co-infections of intestinal schistosomiasis with malaria as well as chronic undernutrition in the form of stunting was also common. Associations between the two parasite infections with anaemia and chronic undernutrition were observed. In view of these findings, Mara region needs to be included in national schistosomiasis and other larger scale parasitic disease control interventions.

## Abbreviations

ANOVA: analysis of variance
BAZ: body mass index for age Z scores
BMI: body mass index
CI: confidence interval
CSPro: census and survey processing system software
EPG: eggs per gram of faeces
HAZ: height for age Z scores
LZIRB: Lake Zone Institutional Review Board
MRCC: Medical Research Coordinating Committee
NIMR: National Institute for Medical Research
OR: odds ratio
STH: soil transmitted helminth infections
SSA: sub-Saharan Africa
WAZ: weight for age Z scores
WHO: World Health Organization
